# Frequency and characteristics of patients with bispectral index values of 60 or higher during the induction and maintenance of general anesthesia with remimazolam

**DOI:** 10.1038/s41598-023-37150-9

**Published:** 2023-06-20

**Authors:** Byung-Moon Choi, Ju-Seung Lee, Kyung Mi Kim, Ji-Yeon Bang, Eun-Kyung Lee, Gyu-Jeong Noh

**Affiliations:** 1 Department of Anaesthesiology and Pain Medicine, Asan Medical Centre, University of Ulsan College of Medicine, 88, Olympic-ro 43-gil, Songpa-gu, Seoul, 05505 Republic of Korea; 2 Department of Statistics, Ewha Womans University, Seoul, South Korea; 3 Department of Anaesthesiology and Pain Medicine and Department of Clinical Pharmacology and Therapeutics, Asan Medical Centre, University of Ulsan College of Medicine, Seoul, Republic of Korea

**Keywords:** Surgery, Clinical trials

## Abstract

In Korea, the approved anesthetic regimen of remimazolam starts with 6 mg/kg/h or 12 mg/kg/h until loss of consciousness, followed by maintenance at 1–2 mg/kg/h. Some patients receiving remimazolam for general anesthesia experience occasional difficulty maintaining bispectral index (BIS) value ˂ 60. This retrospective study aimed to analyze the data from patients undergoing elective surgery under remimazolam based-general anesthesia to determine the frequency and physical characteristics of patients with BIS values ˂ 60. The criterion was established for patients with a poorly maintained BIS value < 60. The frequency and physical characteristics of patients who satisfied this criterion were investigated through their medical records. The modified Brice interview was conducted within 24 h after surgery. Among the 1500 patients included in the analysis, 61 (4.1%) met the criteria for BIS ˂ 60. Based on the modified Brice interview, none of the patients with poorly maintained BIS ˂ 60 complained of intraoperative awareness based on the modified Brice interview or exhibit specific physical characteristics. These patients accounted for less than 5% of the total population studied. Notably, physical characteristics alone are insufficient to predict such patients before surgery.

## Introduction

Remimazolam is a new intravenous benzodiazepine agonist that combines the pharmacological properties of midazolam with the metabolic properties of remifentanil^1^. It shows a small steady-state volume of distribution (35.4 ± 4.2 L, mean ± standard deviation [SD]), high clearance (1.15 ± 0.12 L/min,) and short terminal half-life (70 ± 10 min)^2^. The simulated context-sensitive halftime after an infusion of 4 h is 6.8 ± 2.4 min^2^. Remimazolam is rapidly and extensively metabolized by tissue esterase to a pharmacologically inactive carboxy acid metabolite^3^. In Korea, remimazolam is being used for general anesthesia as a hypnotic agent in the clinical field, having been approved by the Ministry of Food and Drug Safety (MFDS) for sedation and general anesthesia in 2021. The approved anesthetic induction dose of remimazolam is 6 or 12 mg/kg/h until loss of consciousness occurs, and thereafter, it is maintained at 1–2 mg/kg/h. This regimen was determined based on the results of a phase-3 study on remimazolam from Japan^1^.

In general, hypnotic agents, including propofol, are titrated during general anesthesia by referring to a hypnotic depth indicator, such as the bispectral index (BIS™, Medtronic, Dublin, Ireland)^4–6^. The BIS value should be maintained between 40 and 60 during the maintenance of general anesthesia^7,8^. This is because if the BIS value is kept higher than 60, awareness during surgery may occur^9,10^. However, among patients using remimazolam for the induction and maintenance of general anaesthesia, patients whose BIS value could not be maintained at < 60 were occasionally observed despite administration of the maximum allowed dose. Identifying the approximate frequency of these patients may be helpful for anesthesiologists who wish to administer remimazolam for general anesthesia. Whether or not the physical characteristics of these patients differ from those whose BIS values are well maintained at < 60 should be examined.

Therefore, the aim of this study was to retrospectively analyze the data of patients under general anesthesia with remimazolam to determine the frequency and physical characteristics of patients whose BIS values were not maintained at < 60 during the induction or maintenance of anesthesia.

## Materials and methods

### Study design

This was a retrospective study involving patients who had undergone elective surgery under general anesthesia with remimazolam between October 2021 and January 2022 at the Asan Medical Centre (Seoul, Korea). The study protocol was approved by the institutional review board of Asan Medical Centre (approval number: 2022-0298; approval date: March 7, 2022), and the requirement of obtaining informed consent was waived. This study was also registered on an international clinical trials registry platform (http://cris.nih.go.kr, KCT0007101, principal investigator: Byung-Moon Choi., date of registration: 21 March 2022). The primary endpoint, data collection variables, and statistical analysis methods were finalized before accessing the data. This study complied with the Strengthening the Reporting of Observational Studies in Epidemiology (STROBE) statement^11^ and was conducted in accordance with the Declaration of Helsinki.

### Selection criteria

Patients aged 19 years and older who had undergone elective general surgery (stomach, colorectal, breast, hepatobiliary, thyroid, or vascular surgery) were eligible for analyses. In addition, those who had undergone elective surgery for induction and maintenance of anesthesia using remimazolam were included. Patients for whom anesthesia was induced or maintained with hypnotics other than remimazolam were excluded. Since this was a retrospective observational study, no sample size calculations were performed. However, all patients who met the inclusion and exclusion criteria during the defined period for patient identification were included.

### Anesthesia method

The induction and maintenance of anesthesia were performed in accordance with the standard operating procedure at the Asan Medical Centre^12^. Remimazolam was used as the hypnotic agent, and remifentanil was used as the analgesic agent. Remimazolam was titrated to maintain a BIS value of 40–60 during the maintenance of anesthesia. It was administered via a target-controlled infusion using the Minto model^13^. The target effect-site concentration was adjusted within a range of 2 to 20 ng/mL to maintain stable hemodynamic parameters (systolic blood pressure > 80 mmHg; heart rate > 45 beats/min). Rocuronium was administered to maintain a moderate neuromuscular blockade (train-of-four 1 to 2) during surgery. During the surgery, the core temperature was monitored with the noninvasive temperature monitoring system (3 M™ Bair Hugger™ Temperature Monitoring System, St Paul, MN). After the end of surgery, a single intravenous bolus dose of flumazenil (0.2 mg) was administered for the reversal of the effects of remimazolam.

### Criteria for patients with a poorly maintained BIS value < 60 during the induction or maintenance of general anesthesia with remimazolam

**Figure 1:**
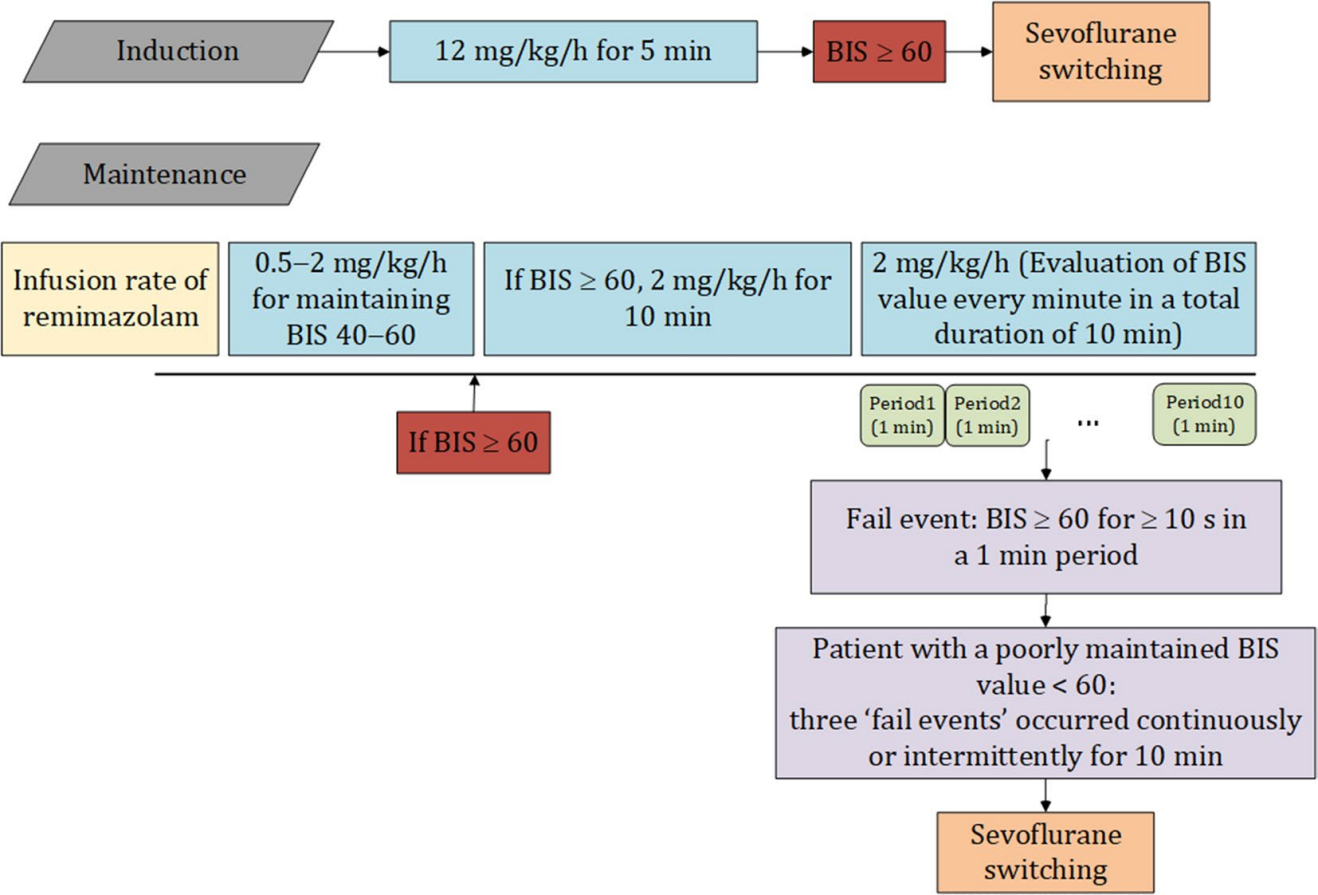
Criteria for patients with poorly maintained bispectral index < 60 during the induction or maintenance of general anesthesia.

Figure 1 summarizes the criteria for patients with a poorly maintained BIS value < 60.

- Induction
  When the BIS value was not maintained at < 60 despite the administration of remimazolam at 12 mg/kg/h for 5 min.

- Maintenance
  When the BIS value was ≥ 60 during the maintenance of anesthesia, the infusion rate of remimazolam was increased to 2 mg/kg/h and maintained for 10 min. Subsequently, the BIS value was observed at periods of 1 min. When the BIS value was maintained at ≥ 60 for 10 s or more in 1 min, it was regarded as a “fail event”. A patient was determined as having a poorly maintained BIS value < 60 when three “fail events” occurred continuously or intermittently for 10 min. The infusion rate of remimazolam was maintained at 2 mg/kg/h until the patient was determined as having a poorly maintained BIS value.

### Course of action for a patient with a poorly maintained BIS value < 60

When a patient with a poorly maintained BIS value < 60 was encountered, the vaporizer of sevoflurane was set to a 2.5% volume, and the anesthetic was switched to sevoflurane. When evaluating whether or not a “fail event” occurred, the occurrence of the signs of inadequate anesthesia was also recorded in the electronic anesthesia record^14^. Because completely ruling out the possibility of awareness was difficult in these patients, the modified Brice interview was conducted within 24 h after surgery according to the usual clinical practice^15,16^.

### Primary and secondary endpoints

The primary endpoint of this study was the evaluation of the incidence of poorly maintained BIS values < 60 among patients under general anesthesia with remimazolam. The secondary endpoint was the comparison of the body characteristics of patients with well-maintained BIS values < 60 and those with poorly maintained BIS values.

### Data collection for the secondary variable analysis

Patient demographic and clinical characteristics, including weight, age, sex, and presence of comorbidities, were manually collected from electronic medical records (EMRs). Data for the most recently examined hemoglobin, albumin, and estimated glomerular filtration rate (eGFR) calculated using the Chronic Kidney Disease Epidemiology Collaboration (CKD-EPI) before surgery was also collected^17^.

### Statistical analysis

Statistical analyses were conducted using the R software (version 4.1.3, R Foundation for Statistical Computing, Vienna, Austria). Data were expressed as mean ± SD or median (25–75%) for continuous variables and count (percentage) for categorical variables. Patient characteristics were compared using the two-sample *t*-test or χ^2^ test, as appropriate. A randomization test was performed to resolve the serious imbalance between the well- and the poorly-maintained groups with BIS values < 60 during anesthesia^18^. A total of 1,500 patients were randomly assigned to two groups of 1,439 and 61 patients. First, a two-sample *t*-statistic for the continuous variable and χ^2^ statistic for the categorical variable were recalculated with this permutation dataset. Subsequently, this process was performed 500,000 times. Among the 500,000 *t*-statistics (t*), the ratio of absolute values larger than the absolute value of the *t*-statistic calculated with the original dataset (t) was used as the two-sided *P*-value of the randomization test. For the categorical variable, among the 500,000 χ^2^ statistics (χ^2^*), the ratio of values larger than the χ^2^ statistic calculated with the original dataset (χ^2^) was used as the *P*-value of the randomization test.

$$ \begin{gathered} {\text{Continuous}}\;{\text{variable}}:\;P{\text{ - value}} = \frac{{\# \left\{ {\left| {t^{*} } \right| > \left| t \right|} \right\}}}{500,000} \hfill \\ {\text{Categorical variable}}: \, \;P{\text{ - value}} = \frac{{\# \left\{ {\left| {\chi^{{2^{*} }} } \right| > \chi^{2} } \right\}}}{500,000} \hfill \\ \end{gathered} $$

A *P*-value < 0.05 was considered to indicate statistical significance.

## Results

**Figure 2:**
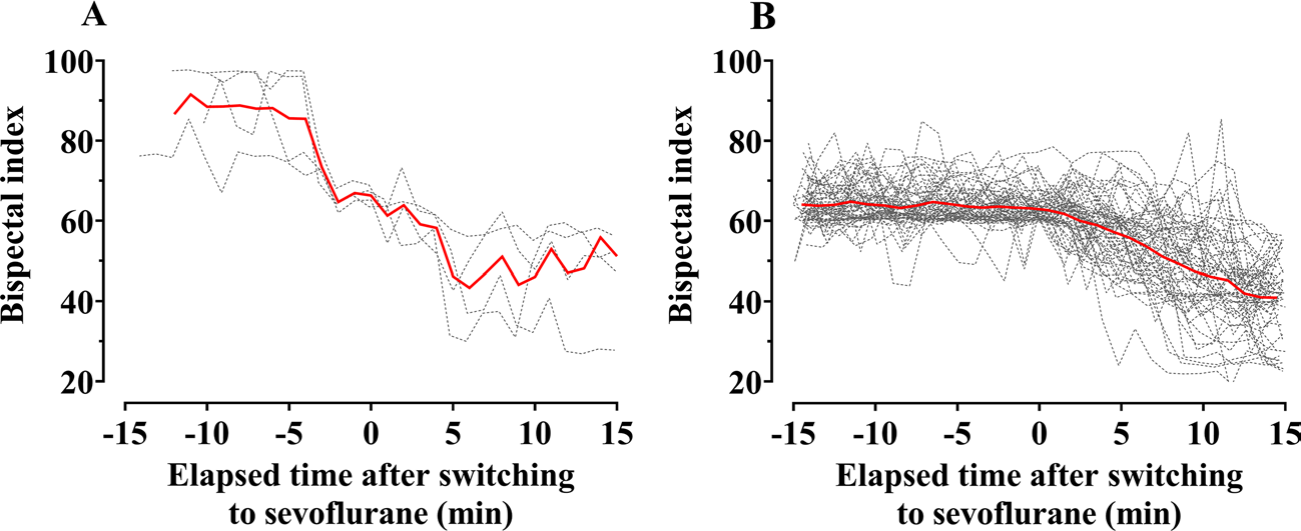
Time courses of the bispectral index (BIS) for 15 min before treatment and after switching from remimazolam to sevoflurane in patients with poorly maintained BIS values < 60 during induction (**A**, n = 4) and maintenance (**B**, n = 56) of anesthesia. The solid red line represents the mean, while the dotted grey line represents individual patient’s BIS value. Among the 57 patients with poorly maintained BIS values < 60 during the maintenance of anesthesia, data for one patient could not be recorded.

**Figure 3:**
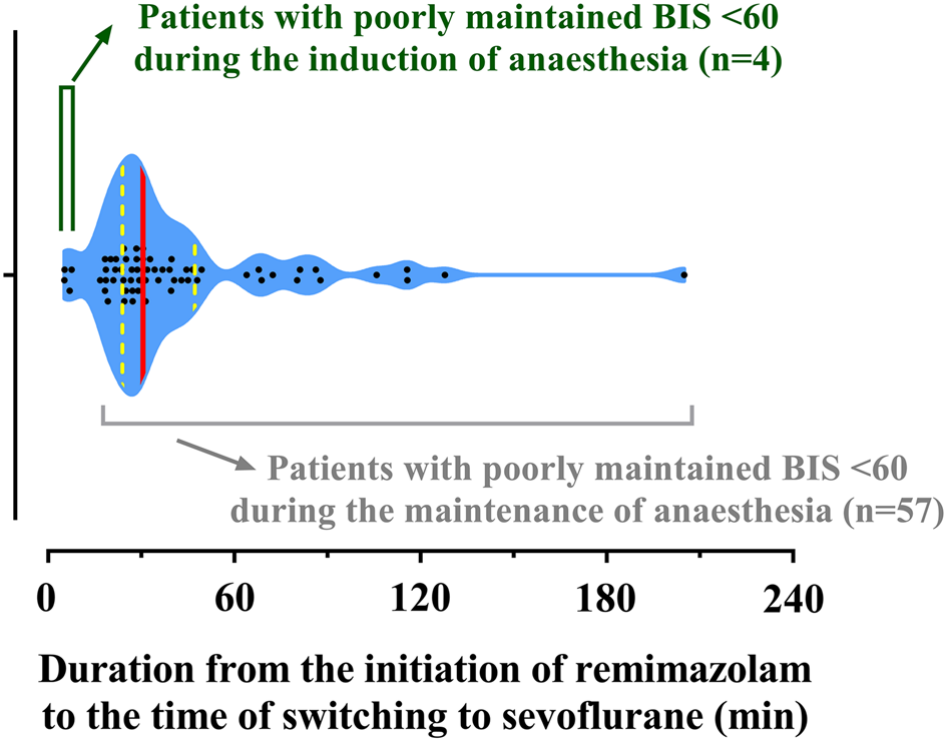
Distribution of the duration from the initiation of remimazolam to the time of switching to sevoflurane. The data are represented by a violin plot. The solid vertical red line represents the median, while the dotted vertical yellow lines represent the first and third quartiles. Black-filled circles represent individual patient values (n = 61).

After applying the inclusion and exclusion criteria, a total of 1,500 patients were included in the analysis. Among them, the number of patients who experienced failure to induction (n = 4) or maintenance of anesthesia (n = 57) was 61 (4.1%). Figure 2 shows the time courses of BIS for 15 min before the procedure and after switching from remimazolam to sevoflurane in patients with poorly maintained BIS value < 60 during general anesthesia. On average, the BIS value remained at < 60 after 5 min of switching to sevoflurane. Figure 3 shows the distribution of duration from the initiation of remimazolam to the time of switching to sevoflurane. Approximately 80% of the patients were switched to sevoflurane within 1 h after starting induction of anesthesia with remimazolam. Table 1 summarizes the physical characteristics of patients with well- and poorly-maintained BIS values < 60. No variables differed significantly between the two groups. Patients whose BIS values were poorly maintained at < 60 had no specific physical characteristics. The modified Brice interview was conducted for all except one patient who had been discharged early among the 57 patients with poorly maintained BIS values < 60 during the maintenance of anesthesia. No one experienced awareness during surgery. In these 57 patients, the median (25–75%) effect-site concentration of remifentanil at the time of the change of the anesthetic to sevoflurane was 7 (5–10.5) ng/mL. Among them, signs of inadequate anesthesia were observed in nine (12.3%) patients.

**Table 1:** Characteristics of patients with well-maintained (BIS ≤ 60) and poorly maintained (BIS > 60) BIS values during general anesthesia.

	BIS ≤ 60 (n = 1,439)	BIS > 60 (n = 61)	*P*-value (*t*-test, Mann–Whitney rank sum test or χ^2^ test)	*P-*value (Randomization test)
Age, years	58.1 ± 13.1	56.1 ± 17.2	0.3804	0.3804
Weight, kg	63.1 ± 11.3	61.3 ± 10.6	0.1998	0.2008
Height, cm	162.1 ± 8.4	161.5 ± 8.1	0.6263	0.6272
BMI, kg/m^2^	24.0 ± 3.6	23.4 ± 3.3	0.2246	0.2256
FFM, kg	44.2 ± 9.2	42.9 ± 9.0	0.2773	0.2773
Male/Female	614/825	23/38	0.5248	0.4290
Hemoglobin, g/dl	13.0 (11.9–14.1)	12.8 (11.1–13.7)	0.079	0.0763
Albumin, g/dl	4.0 (3.7–4.2)	3.9 (3.7–4.2)	0.997	0.8021
eGFR, mL/min/1.73 m^2^	90.8 ± 17.9	90.4 ± 20.7	0.8941	0.8945
ASA PS				
1	190 (13.2)	8 (13.1)	1.0	0.8465
2	1125 (78.2)	50 (82.0)	0.5860	0.4266
3	124 (8.6)	3 (4.9)	0.4344	0.2333
Social characteristics				
Smoking	203 (14.1)	9 (14.8)	1.0	0.8555
Alcohol	472 (32.8)	23 (37.7)	0.5100	0.4080
Comorbidities* (multiple answers possible)				
HTN	467 (32.5)	17 (27.9)	0.5416	0.4013
DM	221 (15.4)	10 (16.4)	0.9694	0.7192
Hyperlipidemia	234 (16.3)	10 (16.4)	1.0	0.8598

Data are expressed as mean ± standard deviation, median (25–75%), or count (percentage). BIS: bispectral index, BMI: body mass index, FFM: fat free mass, ASA PS: American Society of Anesthesiologists Physical Status, eGFR: estimated glomerular filtration rate (calculated using the Chronic Kidney Disease Epidemiology Collaboration [CKD-EPI] creatinine equation), HTN: hypertension, DM: diabetes mellitus.

*Among patients whose BIS were poorly maintained ˂ 60, comorbidities with a frequency of 5% or more are summarized.

## Discussion

Patients with poorly maintained BIS values < 60 constituted < 5% of all surgical patients. These patients had no specific physical characteristics. Based on the modified Brice interview, none of these patients experienced awareness during the surgery.

Underdosing might be the reason why BIS is poorly maintained at < 60 during general anesthesia. However, in the present study, remimazolam was administered at the maximum dose allowed by the Korean MFDS in the process of inducing and maintaining anesthesia. In addition, if remimazolam is administered with this regime, it is not administered at a dosage less than that of the pending approval in Europe. A multicentered clinical trial (phase 3) led by the SURE-TIVA trial group was conducted in Europe to use remimazolam as a general anesthetic. Although the results have not been published, the regimen used in this trial is awaiting approval. The dosing regimen used in the SURE-TIVA trial did not consider patients’ body weight. Remimazolam was administered at 6 mg/min for the first 3 min and then at 2.5 mg/min for the next 7 min. Endotracheal intubation was performed 10 min after the start of administration and maintained at 1.5 mg/min until 10–15 min after the skin incision. Subsequently, it was administered within the range of 0.7–2.5 mg/min. Assuming that the skin incision was started 30 min after remimazolam administration, the maximum dose administered for 60 min was calculated to be 130.5 mg. If this is calculated as the maximum dose of the regimen approved by the Korean MFDS, it is 113.3 mg at 40 kg, 141.7 mg at 50 kg, 170 mg at 60 kg, and 198.3 mg at 70 kg. When the patient’s weight exceeds 47 kg, the maximum dose of remimazolam is always higher when administered with the regimen approved in Korea than with the regimen used in the SURE-TIV trial. Considering that the average body weight (SD) of patients with poorly maintained BIS values < 60 in this study was 61.3 (10.6) kg, underdosing is unlikely to be the reason.

BIS may poorly reflect the central nervous system effect of remimazolam. The algorithm of BIS was developed based on 5,000 h of electroencephalogram (EEG) data collected through various anesthesia regimens from approximately 1500 participants^19^. BIS was well validated for various anesthetics^20^, but remimazolam EEG data were not included when developing the algorithm of BIS. Midazolam EEG data may have been included, but the characteristics of remimazolam as a soft drug were not reflected in the algorithm development process. Although the algorithm of BIS has not been fully disclosed, it consists of four sub-parameters (burst suppression ratio, QUAZI suppression, relative β-ratio, and SyncFastSlow)^19^. The weight values of these four sub-parameters change according to the hypnotic depth, but how these weights are reflected is not disclosed. In the patient’s brain, sufficient unconsciousness required for general anesthesia was induced but not properly reflected in the sub-parameters; thus, considering that the BIS value will be > 60 may be possible. Even if remimazolam was continuously administered at the maximum permitted infusion rate, the suppression ratio presented in the BIS monitor was zero in most patients. This may suggest that remimazolam may have a slightly weaker effect on lowering BIS compared to propofol. However, this is only a speculation, and the exact cause may be revealed only through a precise EEG analysis involving participants receiving remimazolam at the maximum infusion rate.

The possibility that the degree of muscle relaxation may have affected the BIS value can be considered. In a previous study, the effect of neuromuscular blocking agents on muscle activity was evaluated in intensive care unit patients^21^. After the administration of atracurium (0.5 mg/kg), the BIS value significantly decreased from an average of 67 to 43 although the level of sedation was the same^21^. In the present study, more than half of the patients whose BIS was not well maintained ˂ 60 were recognized within 30 min after the administration of the rocuronium loading dose (0.8 mg/kg; Fig. 3), indicating that muscle relaxation was well maintained. The remaining patients also received an additional dose of rocuronium to maintain the TOF count below two, and muscle relaxation was maintained to some extent. To date, no study has shown a significant difference in the BIS value between TOF counts of two and zero. Clinically, when the TOF count is two, it becomes zero after an additional administration of rocuronium, and the BIS values seem to be almost similar. Since more than 70% of nicotinic acetylcholine receptors were blocked with the TOF count was two^22^, the effect of electromyographic activity that can increase the BIS value can be considered insignificant. The degree of muscle relaxation was similar to that of patients whose BIS was well maintained ˂ 60 after the administration of remimazolam.

The possibility that endozepines may have affected the BIS value should also be considered. Endozepines are endogenous benzodiazepine ligands that act as positive allosteric modulators of the GABAA receptor and perform the same action on the central nervous system as exogenous benzodiazepines^23,24^. Clinically, they have been linked to hepatic encephalopathy and, controversially, to some cases of recurrent stupor^25^. Initially, the key diagnostic test is stupor, which is sensitive to the benzodiazepine receptor antagonist flumazenil in the absence of exogenous benzodiazepines. Clinical findings related to endozepines are mainly related to flumazenil. For instance, the administration of flumazenil can induce panic attacks in patients with panic disorder but not in healthy controls^26^. Endozepines were not considered in this study. However, for patients with poorly maintained BIS ˂ 60, endozepines may not have exerted a significant effect. This is because endozepines act to decrease the BIS value. Endozepines levels could be extremely low in these patients. However, when remimazolam is administered externally at the approved maximum infusion rate, the BIS value should be maintained ˂ 60 regardless of endozepines levels. In a case report, two patients receiving benzodiazepines before surgery required large doses of remimazolam to achieve a BIS of less than 60^27^. This suggests that the patient’s concomitant medications may affect intraoperative BIS values. Table S1 summarizes the medications taken before surgery by patients whose BIS was poorly maintained ˂ 60. None of these patients were receiving benzodiazepines. Moreover, none of the patients who took psychologic medications showed altered BIS values during general anesthesia. In a previous study, the interaction between remimazolam and albumin was studied, and the formation of a 1:1 complex of remimazolam–human serum albumin was confirmed^28^. This suggests that the effect of remimazolam may vary depending on the albumin level. However, in the present study, the two groups did not differ significantly in the albumin or hemoglobin concentration measured before surgery (See Table 1).

In this study, most patients with a poorly maintained BIS value < 60 had a BIS value > 60 for at least 10 min. The modified Brice interview is the preferred modality for assessing intraoperative awareness with explicit recall^29^. The main reason for the absence of awareness in this study was probably the anterograde amnesia effect of remimazolam. Alternatively, in reality, an appropriate level of unconsciousness for general anesthesia was maintained, but BIS’s algorithm did not reflect this well; thus, it might be more than 60 numerically. Further research is necessary to determine whether or not memory formation occurs even if the BIS value is ≥ 60 when general anesthesia is induced with remimazolam. In addition, whether or not explicit and implicit memories have occurred should be determined. The occurrence of implicit memory can be evaluated using the process dissociation procedure^30^, but it could not be evaluated here because this was a retrospective study. As knowledge from additional research results is accumulated, the criterion for an adequate hypnotic depth may be set at a BIS value > 60 when undergoing general anesthesia with remimazolam. However, at this point, the BIS value should be maintained at < 60.

The fact that the physical characteristics of the patients with a poorly maintained BIS value of < 60 were unremarkable suggests that it is not possible to predict in advance that the BIS will not be maintained well before surgery. However, it may be possible to explain the difference in metabolism. Remimazolam is rapidly hydrolyzed in the body by tissue esterases (chiefly liver carboxylesterase) to the inactive carboxylic acid (CNS 7054)^3,31^. Several studies have been conducted on the pharmacogenetics of carboxylesterases^32,33^, and genetic variants of the carboxylesterase genes can affect drug metabolism and clinical outcomes^32^. To date, few studies have investigated the pharmacogenetics of remimazolam; thus, research on this is also required.

## Conclusion

Of the 1,500 patients included in the analysis, 61 (4.1%) met the criteria for poorly maintained BIS values of < 60. Approximately 80% of patients with poorly maintained BIS values of < 60 were observed within 1 h of initiating the remimazolam administration. The modified Brice interview showed that none of the patients with a poorly maintained BIS value < 60 complained of intraoperative awareness. Patients whose BIS values were poorly maintained < 60 developed unremarkable physical characteristics.

## Supplementary Information

Supplementary Table S1.

## Data Availability

The datasets generated during and/or analyzed during the current study are available from the corresponding author on reasonable request.
