# Supplementary material for: Frequency and characteristics of patients with bispectral index values of 60 or higher during the induction and maintenance of general anesthesia with remimazolam
Source: Sci Rep. 2023 Jun 20;13:9992. doi: 10.1038/s41598-023-37150-9 (PMC10282058; doi:10.1038/s41598-023-37150-9)
Supplement: Supplementary file 1 — Supplementary Table S1. [file 41598_2023_37150_MOESM1_ESM.docx]

**Table S1**. Concurrent medications of patients (n = 61) whose intraoperative bispectral index values were poorly maintained below 60

| Category | Reason | Frequency (%) | Drug name |
| --- | --- | --- | --- |
| Cardiovascular | Hypertension | 17 (27.9) | Amlodipine, telmisartan, carvedilol, propranolol, olmesartan, candesartan, losartan |
|  | Hyperlipidemia | 10 (16.3) | Rosuvastatin, simvastatin, atorvastatin, lovastatin |
|  | Anti-platelet agent | 4 (6.6) | Clopidogrel, sarpogrelate |
| Endocrinology | Diabetes mellitus | 10 (16.3) | Metformin, glimepiride, linagliptin, empagliflozin, pioglitazone |
| Gastrointestinal | Antacid | 7 (11.5) | Rabeprazole, esomeprazole, lansoprazole, almagate |
|  | Gastroprokinetic | 5 (8.2) | Mosapride |
|  | Gastroprotective | 6 (9.8) | Rebamipide, lafutidine, teprenone |
|  | Antispasmodic | 3 (4.9) | Alverine, tiropramide |
| Urinary | Prostate hyperplasia | 4 (6.6) | Silondosil, tamsulosin |
|  | Overactive bladder | 3 (4.9) | Mirabegron |

Concurrent medications with a frequency of three (4.9%) or more patients are summarized.
